# Nutritional and health status among nursing home residents in Lebanon: comparison across gender in a national cross sectional study

**DOI:** 10.1186/1471-2458-14-629

**Published:** 2014-06-20

**Authors:** Jacqueline H Doumit, Ramzi N Nasser, Dimitri R Hanna

**Affiliations:** 1 Nursing and Health Sciences Department, Notre Dame University-Louaize, Zouk Mosbeh, Lebanon; 2 College of Education, Qatar University, Doha, Qatar and Research and Development Institute-International, Byblos, Lebanon; 3 Research and Development Institute-International, Byblos, Lebanon

**Keywords:** Gender differences, Nutritional status, Mini-Nutritional Assessment, Long-term care, Elderly, Aging, Nursing homes, Lebanon

## Abstract

**Background:**

This study described the differences between elderly men and women living in Lebanese long-term care nursing homes on socio-economic, health and nutritional status.

**Methods:**

This study used a cross-sectional design. Field researchers obtained data from 221 residents; 148 (67%) women and 73 (33%) men, living in 36 nursing homes. Data on health conditions; nutritional, psychological, and functional status; socio-demographic characteristics, as well as social relations were collected. The analysis used both chi-square and t-test tests.

**Results:**

The majority of elderly had low socio-economic and poor health status. In comparison to men, women were significantly less educated, had lower occupational status, had no partner, relied financially on their children and relatives, and enjoyed better social relations and health behaviours. Furthermore, the prevalence of both; malnutrition, and at risk of malnutrition, were at 3.2% and 27.6% respectively. There was no statistically significant difference between women and men on Mini Nutritional Assessment, Activities of Daily Living, Geriatric Depression Scale, Body Mass Index, and chronic diseases. While women reported “good” health status compared to men, they continued to have higher prevalence of diseases and chronic pain.

**Conclusions:**

This study explored the socio-demographic, health, and nutritional status of elderly residing in Lebanese nursing homes and compared these characteristics across gender. The results indicated the need of health support and institutional interventions for elderly women residents.

## Background

Worldwide, the old population has been growing rapidly [1]. There has been a strong positive relation between age and cognitive functioning, age and functional impairment, and age and malnutrition [2]. Studies have suggested that older age individuals tended to suffer from a large number of psychological, functional and health related diseases because of environmental living conditions [2].

Differences between elderly men and women have been reported in the literature. Women lived longer than men. They were more likely to suffer from malnutrition [3,4] and to acquire fatal illnesses such as arthritis, chronic back pain, asthma and anemia rendering them more dependent in their daily lives [5]. In Lebanon and the Middle East, women were more likely to have lower socioeconomic status and to be either widowed or single and malnourished [6-11] than men. Differences between institutionalized and non-institutionalized elderly have also been reported. The findings generally showed that all elderly living in institutions [3,12,13], and women in particular [4], tended to suffer from high prevalence of malnutrition.

Lebanon, a small middle-income country in the Middle East region, experienced a rapid increase in the elderly population aged 65 years and above from 7.4% in 2004 [14] to 9.6% in 2010 [15]. In parallel, the number of long-term care nursing homes (NHs) and their elderly population increased from 33 NHs with 2,660 elderly in 2005 [16] to 46 NHs with 3,299 elderly in 2008 [17]. Currently, there are 49 NHs with approximately 4,000 elderly [18] distributed across the country and concentrated largely in urban areas. Nasser and Doumit [19] identified three types of NHs in Lebanon: 1) those NHs that received independent elders, 2) those that received dependent or sick elders and 3) those that received all types of elders.

In reviewing the literature on elderly in Lebanon, few studies did focus on elderly residing in NHs [19-25] and none whatsoever did examine at the national level the socio-demographic and health characteristics across gender. This study came to fill the gap in the Lebanese elderly literature. It compared elderly men and women residing in NHs on demographic, socio-economic, functional, psychological and nutritional status.

## Methods

### Study design and setting

This study used a questionnaire targeting all elderly people living in Lebanese NHs. It was conducted in 2007–2009. All administrators of 36 NHs showed their willingness to participate and signed an informed consent. At that time, an ethics committee did not exist at both the institutional and national levels. Instead, we turned to the Ethics Review Committee of the World Health Organization, got their approval for the research methodology and to carry out the study. We also sought the approval of Public Health and Social Affairs Ministries as well as the National Association of Elderly Affairs. When we commenced the data collection, field researchers entered NHs, described the project to each elderly resident and expressed not only their need for information but also their respect for confidentiality and information privacy. Our researchers were clear about the voluntary aspect of the study and requested verbal approval from each elderly before they started interviewing. The exclusion of elderly from the study began with the screening of medical dossiers.

### Sample and exclusion criteria

**Figure 1:**
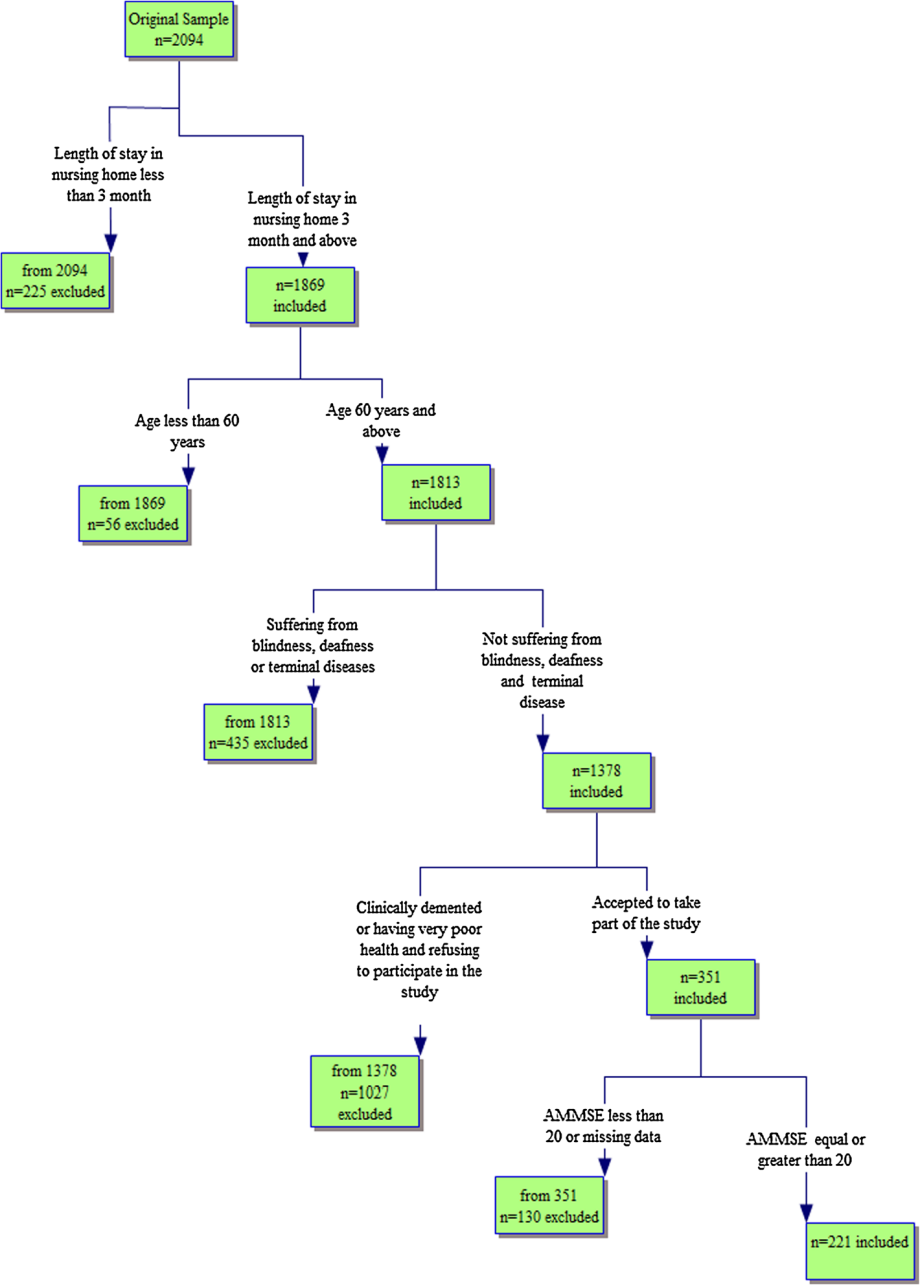
Sample selection flow chart.

Five exclusion criteria resulted in the inclusion of those elderly who were able to interact with interviewers. These criteria were: 1) having been in NHs for less than three months; 2) being under 60 years of age; 3) suffering from a terminal disease; 4) being blind and/or deaf; 5) clinically demented or of very poor heath and refusing to participate in this study and 6) exhibiting cognitive functioning measured by a score of less than 20 for the Adapted Mini Mental State Examination (AMMSE) [21]. Figure 1 presented the selection sample- flow chart. As a result, the study was left with only 354 out of the original 2,094 elderly living in the 36 NHs. In total 221 elderly had valid responses (without missing information) with the number of women n = 148 (67%) twice of men n = 73 (33%).

### Assessment tools

The following instruments, described by Doumit and Nasser [21], were used in this study:

1) The AMMSE, an Arabic adapted version of the Mini Mental State Examination (MMSE) [21], was used to evaluate cognitive functioning. It made the questions simple and easy to follow among illiterate elderly. Changes were made to those items that demanded writing, reading and arithmetical knowledge. All respondents who scored less than 20 on the AMMSE were excluded from the study.

2) The Geriatric Depression Rating Scale (GDS) Arabic version [26] provided measures of depressive symptoms among elderly [27]. A score between 11 and 15 indicated severe depression, while between 6 and 10, mild to moderate depression and equal and less than 5 (GDS ≤ 5), a normal person with no depression.

3) The Activities of Daily Living (ADL) Arabic version [28] provided information about individual ability to perform basic functioning tasks [29]. The components of this index were highly ordered which allowed for the loss of independence to occur gradually in the order of bathing, dressing, going to the toilet, movement, continence, eating and drinking. The response format for each of the components was 0, half and 1. A score of 0 on each component meant dependence; a half indicated that the elderly were partially independent while 1 meant independence. The ADL score was from 0–6 with the cut-off points: ≤2 for severe physical disability, 2.5 to 5.5 for moderate disability and 6 for complete independence.

4) The Mini Nutritional Assessment (MNA) assessed the nutritional status among elderly [30,31]. The Full form of the MNA was administered in two steps: screening and then assessment. The first screening step used the MNA-short form (MNA-SF) which provided a 6-item checklist dealing with appetite, weight loss, mobility, current illness, neuropsychological problems and body mass index (BMI). The MNA-SF maximum score was 14. If the screening score on the MNA-SF was12 points or greater, the elderly was classified as well-nourished and there was no need to complete the assessment. Otherwise, with 11 points or lower, the person was required to complete the assessment part of the Full MNA. The Full MNA maximum score was 30. Elderly receiving < 17 points score indicated that the respondent was malnourished, between 17 and 23.5 indicated that the respondent was at risk of malnutrition and more than 24 (≥24) indicated that the elderly was well-nourished.

### Interview and medical dossier

Participants were also interviewed to solicit information on demographics, socio-economic levels, oral health status and smoking behaviors. The educational level was classified as follows: 1) low; included those either illiterates or had not completed elementary school, 2) intermediate; included those who had not terminated secondary school and 3) high; included those having a university degree.

Two main measures of socio-economic status were considered for the study: employment of elderly and occupational status. Elderly answered whether they were employed or not and for occupational status; rated whether they classified themselves as: 1 = “low income job, farmer, owner a small shop, or vocational job”; 2 = “middle income job, owner middle size farm or middle level businessman” and 3 = “high income job, a white-collar job or owner of a large business and tradesman.” “Yes” and “No” questions provided information on whether the elderly was either a smoker or non-smoker and had or had not an oral health problems.

Data on prescribed medication and nutritional supplements intake was obtained from the elderly resident dossier. Self-rated health (SRH) was assessed by asking the elderly resident to rate their overall health on a 4-point scale as excellent, very good, good and poor. Those reporting excellent, very good and good health were considered as having “good” self-rated health. In addition, to measure social relation, each elderly was asked these questions: 1) “Do you visit your family members, relatives, friends and colleagues?” and 2) “Do they visit you at your place? In this study, a social relation was considered positive when the elderly replied positively to either one of these two questions.

### Anthropometric measurements

Weight was measured by an electronic digital scale from Eagle Company - China (model #ELP 300 L) with light clothing and no shoes to the nearest 0.1 kg. Height (H) was also measured in standing position without shoes using a wall mounted height meter from Ruimax Company - China. Feet were put together with heels, buttocks, shoulder and back of the head touching the wall and the head was held in Frankfurt plane position. The measurement precision was to the nearest 0.1 cm. For those elders who were not able to stand, height was calculated using the demispan formula provided by Nestlé Nutrition Institute (for women: H(cm) = (1.35 × demispan (cm)) + 60.1; for men: H(cm) = (1.4 × demispan (cm)) + 57.8) [32]. The Body Mass Index (BMI) of each elder was calculated by the (BMI = weight (kg) divided by the height (m^2^)). The World Health Organization classified adults as obese (BMI > 30), overweight (BMI = 25–29.99) and normal (BMI = 18.50-24.99) [33]. In this study, elderly were considered undernourished or underweight for BMI < 21 along with the French National Authority for Health classification [34]. Mid-arm circumference (MAC) and calf circumference (CC) were measured twice using a flexible measure tape to the nearest 0.1 cm. MAC was measured at half distance between olecranon and acromion at the non-dominant arm and the mean value was recorded. The CC was measured at the widest point of the undressed calf and the largest value was recorded.

### Statistical analysis

Statistical analyses were performed with SPSS (20.0). Nominal variables were presented as absolute numbers and percentages while continuous variables as means and standard deviations (SD). Chi-square tests were performed through a cross tabulation of categorical data and a t-test was used to compare means between genders.

## Results

### Socio-demographic characteristics

Table 1 presented the socio-demographic characteristics of the elderly residents crossed by gender. The study sample included 148 women (67%) and 73 men (33%). The age range of participants was between 75–84 years old with the mean age M = 78.4 (SD = 7.7) years. A high percentage of respondents were living in NHs for more than one year, illiterate or had a low educational level, financially dependent on their children or relatives and not employed. Moreover, they had no partner, no healthcare coverage, and a low status jobs. Note that n = 45 (20%) of women had been working as homemakers prior to their nursing home admission.

**Table 1:** Socio-demographic representation of the elderly sample, distributed by gender

***Variables***	***Women (N = 148)***	***Men (N = 73)***	***Total (N = 221)***	***Chi -square***	***P***
***age, Mean (SD)***	***78.7 (7.5)***	***77.8 (8.1)***	***78.4 (7.7)***		***0.464***
***Age Classification, n (%)***	***145 (66.5%)***	***73 (33.5%)***	***218***	***2.15***	***0.341***
Young – old (60-74Y)	*36 (24.8%)*	*25 (34.2%)*	*61 (28%)*		
Old - Old (75–84 Y)	*76 (52.4%)*	*33 (45.2%)*	*109 (50%)*		
Oldest - Old (≥85 Y)	*33 (22.8%)*	*15 (20.5%)*	*48 (22%)*		
***Length of stay in institution, n (%)***	***147 (67.1%)***	***72 (32.9%)***	***219***	***0.32***	***0.854***
<6 months	*11 (7.5%)*	*5 (6.9%)*	*16 (7.3%)*		
6-12 months	*11 (7.5%)*	*4 (5.6%)*	*15 (6.8%)*		
>12 months	*125 (85%)*	*63 (87.5%)*	*188 (85.8%)*		
Education level*, n (%)	***147 (66.8%)***	***73 (33.2%)***	***220***	***14.58***	***<0.001***
Low	*97 (66.0%)*	*41 (56.2%)*	*138 (62.7%)*		
Intermediate	*46 (31.3%)*	*19 (26%)*	*65 (29.5%)*		
High	*4 (2.7%)*	*13 (17.8%)*	*17 (7.7%)*		
***Marital status, n (%)***	***148 (67.89%)***	***73 (32.11%)***	***221***	***12.34***	***0.006***
Single	*54 (36.5%)*	*31 (42.5%)*	*85 (38.5%)*		
Married	*8 (5.4%)*	*11 (15.1%)*	*19 (8.6%)*		
Widowed	*81 (54.7%)*	*25 (34.2%)*	*106 (48.0%)*		
Divorced	*5 (3.4%)*	*6 (8.2%)*	*11 (5.0%)*		
Previous occupation status**, n (%)	***104 (69.3%)***	***46 (30.7%)***	***150***	***34***	***<0.001***
Low	*53 (65.4%)*	*15 (21.7%)*	*68 (45.3%)*		
Intermediate	*21 (25.9%)*	*24 (34.8%)*	*45 (30.0%)*		
High	*7 (8.6%)*	*30 (43.5%)*	*37 (24.7%)*		
***Currently working in nursing homes, n (%)***	***148 (67.89%)***	***73 (32.11%)***	***221***	***2.45***	***0.118***
Yes	*26 (17.6%)*	*7 (9.6%)*	*33 (14.9%)*		
No	*122 (82.4%)*	*66 (90.4%)*	*188 (85.1%)*		
***Financial dependence, n (%)***	***72 (66.6%)***	***36 (33.4%)***	***108***	***10.18***	***0.001***
Yes	*59 (81.9%)*	*19 (52.8%)*	*78 (72.2%)*		
No	*13 (18.1%)*	*17 (47.2%)*	*30 (27.8%)*		
***Health insurance, n (%)***	***138 (67.6%)***	***66 (32.4%)***	***204***	***0.82***	***0.774***
Yes	*33 (23.9%)*	*17 (25.8%)*	*50 (24.5%)*		
No	*105 (76.1%)*	*49 (74.2%)*	*154 (75.5%)*		

Legend: *The educational level was classified as follows: 1) low; included those either illiterates or had not completed elementary school, 2) intermediate; included those who had not terminated secondary school and 3) high; included those having university degree. **The occupational status was classified as follows: 1) low income job, farmer, owner a small shop, or vocational job, 2) middle income job, owner middle size farm or middle level businessman and 3) high income job, a white-collar job or owner of a large business and tradesman.

There were no significant differences between elderly men and women on; length of stay in the NHs, current work status, and healthcare coverage. In comparison to men, women were significantly more likely to be less educated, to have low occupational status and to depend financially on their children or others. However, men were more likely to be married than women.

### Nutritional status and health characteristics

Table 2 presented the results of nutritional status and elderly health measures. According to the MNA, n = 153 (69.2%) elderly were well-nourished, n = 61 (27.6%) at risk of malnutrition and n = 7 (3.2%) malnourished. The mean BMI was M = 25.9 (SD = 5.4) and showed that n = 65 (31.1%) of elderly were of normal weight, n = 69 (33%) overweight, n = 42 (20.1%) obese and n = 33 (15.8%) underweight or malnourished. No significant differences were reported between men and women on either the MNA or BMI scores. However, obesity was more prevalent among women but short of reaching statistical significance.

**Table 2:** Health and lifestyle characteristics of the elderly sample, distributed by gender

**Variables**	**Women (N = 148)**	**Men (N = 73)**	**Total (N = 221)**	**Chi -square**	**P**
***MNA, n (%)***	**148 (67.89%)**	**73 (32.11%)**	**221**	**0.22**	**0.89**
Malnutrition (Full MNA < 17)	5 (3.4%)	2 (2.7%)	7 (3.2%)		
At risk of malnutrition (Full MNA:17–23)	42 (28.4%)	19 (26.0%)	61 (27.6%)		
Well-nourished (Full MNA ≥ 24 or MNA-SF ≥ 12)	101 (68.2%)	52 (71.2%)	153 (69.2%)		
***BMI, Mean (SD)***	**26.17 (5.72)**	**25.29 (4.64)**	**25.88 (5.39)**		**0.266**
***BMI classification*, n (%)**	**139 (66.5%)**	**70 (33.5%)**	**209**	**4.84**	**0.184**
Underweight (<21)	23 (16.5%)	10 (14.3%)	33 (15.8%)		
Normal weight (21–24.99)	38 (27.3%)	27 (38.6%)	65 (31.1%)		
Overweight (25–29.99)	45 (32.4%)	24 (34.3%)	69 (33.0%)		
Obese (≥30)	33 (23.7%)	9 (12.9%)	42 (20.1%)		
***Self rated health*, n (%)**	**148 (67.3%)**	**72 (32.7%)**	**220**	**14.5**	**0.002**
Very good*	99 (66.9%)	55 (76.4%)	154 (70.0%)		
Good	42 (28.4%)	9 (12.5%)	51 (23.2%)		
Poor	7 (4.7%)	8 (11.1%)	15 (6.8%)		
***Daily Drug intake*, n (%)**	**148 (67.89%)**	**73 (32.11%)**	**221**	**8.82**	**0.012**
No	12 (8.1%)	16 (21.9%)	28 (12.7%)		
≤3 drugs/day	39 (26.4%)	19 (26.0%)	58 (26.2%)		
>3 drugs/day	97 (65.5%)	38 (52.1%)	135 (61.1%)		
***Insomnia*, n (%)**	**144 (66.7%)**	**72 (33.3%)**	**216**	**1.28**	**0.258**
Yes	101 (70.1%)	45 (62.5%)	146 (67.6%)		
No	43 (29.9%)	27 (37.5%)	70 (32.4%)		
***Number of diseases*, n (%)**	**148 (67.89%)**	**73 (32.11%)**	**221**	**4.39**	**0.111**
No	23 (15.5%)	20 (27.4%)	43 (19.5%)		
≤3	109 (73.6%)	46 (63.0%)	155 (70.1%)		
>3	16 (10.8%)	7 (9.6%)	23 (10.4%)		
***Hospitalization during last year*, n (%)**	**147 (66.82%)**	**73 (33.18%)**	**220**	**0.25**	**0.617**
Yes	43 (29.3%)	19 (26.0%)	62 (28.2%)		
No	104 (70.7%)	54 (74.0%)	158 (71.8%)		
***Nutritional supplements intake*, n (%)**	**148 (67.89%)**	**73 (32.11%)**	**221**	**3.63**	**0.057**
Yes	85 (57.4%)	32 (43.8%)	117 (52.9%)		
No	63 (42.6%)	41 (56.2%)	104 (47.1%)		
***Physical activities*, n (%)**	**148 (67.89%)**	**73 (32.11%)**	**221**	**11.4**	**0.003**
No	88 (59.5%)	29 (39.7%)	117 (52.9%)		
Occasionally	42 (28.4%)	23 (31.5%)	65 (29.4%)		
Regularly	18(12.2%)	21 (28.8%)	39 (17.6%)		
***Current smokers*, n (%)**	**148 (67.89%)**	**73 (32.11%)**	**221**	**11.22**	**0.001**
Yes	31 (20.9%)	31 (42.5%)	62 (28.1%)		
No	117 (79.1%)	42 (57.5%)	159 (71.9%)		
***Chronic pain*, n (%)**	**147 (66.82%)**	**73 (33.18%)**	**220**	**5.47**	**0.019**
Yes	81 (55.1%)	28 (38.4%)	109 (49.5%)		
No	66 (44.9%)	45 (61.6%)	111 (50.5%)		
***Oral health problems*, n (%)**	**148 (67.89%)**	**73 (32.11%)**	**221**	**4.5**	**0.034**
Yes	75 (50.7%)	48 (65.8%)	123 (55.7%)		
No	73 (49.3%)	25 (34.2%)	98 (44.3%)		

Legend: MNA: Mini Nutritional Assessment, BMI: Body mass index. *Elderly who reported excellent and very good health.

Elderly health (SRH), n = 205 (93.2%) was self-rated as “good”, while only n = 15 (6.8%) reported poor health with women being more likely to consider their overall health as “good” in comparison to men.

A high percentage of elderly had chronic diseases, insomnia, chronic pain and oral health problems, were on prescribed medication and nutritional supplements, exhibited nonsmoking behavior and were not physically active.

**Figure 2:**
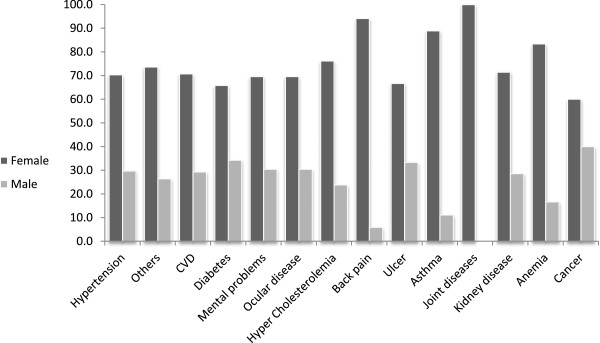
**Prevalence (%) of comorbidities distributed by gender among nursing home residents in Lebanon.** Legend: CVD: Cardiovascular diseases.

Furthermore, there were no significant differences between men and women on insomnia, comorbid illnesses, hospitalization (during last year) and intake of nutritional supplements. However, men were significantly more likely to be physically active than women, to exhibit smoking behaviors and to have oral health problems. In addition, men were significantly less likely to take prescribed medication and to suffer from chronic pain. The most prevalent diseases among elderly were hypertension (46%) followed by cardiovascular diseases (26%), diabetes (18%), mental problems (11%), ocular diseases (11%) and hypercholesterolemia (10%) (Data not shown).In addition, all diseases were more prevalent among women than men however, results were not statistically significant (see Figure 2).

### Functional ability, psychological status and social relations

Table 3 showed that n = 78 (35.3%) of elderly were able to perform the basic activities of daily living. In comparison to women, men reported a higher level of functional ability on all the ADL components except for continence. There were significant differences for bathing and dressing between men and women.

**Table 3:** Functional ability, psychological status and social relations of the elderly sample, distributed by gender

**Variables**	**Women (N = 148)**	**Men (N = 73)**	**Total (N = 221)**	**Chi -square**	**P**
***ADL*, n (%)**	**148 (67.89%)**	**73 (32.11%)**	**221**	**3.78**	**0.151**
Totally independent (=6)	50 (33.8%)	28 (38.4%)	78 (35.3%)		
Moderate disability (2.5-5.5)	74 (50.0%)	40 (54.8%)	114 (51.6%)		
Severe disability (≤2)	24 (16.2%)	5 (6.8%)	29 (13.1%)		
***Continence*, n (%)**	**145 (66.8%)**	**72 (33.2%)**	**217**	**4.92**	**0.086**
Continent	113 (77.9%)	53 (73.6%)	166 (76.5%)		
Partially incontinent	16 (11%)	15 (20.8%)	31 (14.3%)		
Totally incontinent	16 (11%)	4 (5.6%)	20 (9.2%)		
***Mobility*, n (%)**	**148 (67.89%)**	**73 (32.11%)**	**221**	**4.13**	**0.127**
Mobile	103 (69.6%)	59 (80.8%)	162 (73.3%)		
Partially mobile	32 (21.6%)	12 (16.4%)	44 (19.9%)		
Immobile	13 (8.8%)	2 (2.7%)	15 (6.8%)		
***Feed oneself*, n (%)**	**148 (67.89%)**	**73(32.11%)**	**221**	**2.05**	**0.359**
Able	103 (69.6%)	59 (80.8%)	206 (93.2%)		
Partially	32 (21.6%)	12 (16.4%)	11 (5.0%)		
Unable	13 (8.8%)	2 (2.7%)	4 (1.8%)		
***Use the toilet*, n (%)**	**148 (67.3%)**	**72 (32.7%)**	**220**	**3.79**	**0.15**
Able	101 (68.2%)	56 (77.8%)	157 (71.4%)		
Partially	20 (13.5%)	10 (13.9%)	30 (13.6%)		
Unable	27 (18.2%)	6 (8.3%)	33 (15.0%)		
***Dressing*, n (%)**	**148 (67.89%)**	**73 (32.11%)**	**221**	**8.47**	**0.014**
Able	85 (57.4%)	50 (68.5%)	135 (61.1%)		
Partially	19 (12.8%)	14 (19.2%)	33 (14.9%)		
Unable	44 (29.7%)	9 (12.3%)	53 (24.0%)		
***Bathing*, n (%)**	**148 (67.89%)**	**73 (32.11%)**	**221**	**6.18**	**0.045**
Able	58 (39.9%)	34 (46.6%)	93 (42.1%)		
Partially	37 (25.0%)	25 (34.2%)	62 (28.1%)		
Unable	52 (35.1%)	14 (19.2%)	66 (29.9%)		
***GDS*, n (%)**	**148 (67.89%)**	**73 (32.11%)**	**221**	**0.09**	**0.957**
Normal/no depression (≤5)	59 (39.9%)	29 (39.7%)	88 (39.8%)		
Mild/ moderate depression (6–10)	73 (49.3%)	37 (50.7%)	110 (49.8%)		
Severe depression (≥11)	16 (10.8%)	7 (9.6%)	23 (10.4%)		
***Being visited*, n (%)**	**148 (67.89%)**	**73 (32.11%)**	**221**	**5.6**	**0.018**
Yes	130 (87.8%)	55 (75.3%)	185 (83.7%)		
No	18 (12.2%)	18 (24.7%)	36 (16.3%)		
***Visiting family & friends*, n (%)**	**148 (67.89%)**	**73 (32.11%)**	**221**	**0.2**	**0.656**
Yes	49 (33.1%)	22 (30.1%)	71 (32.1%)		
No	99 (66.9%)	51 (69.9%)	150 (67.9%)		
***Social relations*, n (%)**	**148 (67.89%)**	**73 (32.11%)**	**221**	**2.92**	**0.08**
Yes	132 (89.2%)	59 (80.8%)	191 (86.4%)		
No	16 (10.8%)	14 (19.2%)	30 (13.6%)		

Legend: ADL: Activities of Daily Living, GDS: Geriatric Depression Scale.

According to the 15 item GDS score, 60.2% of the sample showed depressive symptoms. There were no significant differences among men and women on GDS scores and on social relations. However, significant differences were shown on social relation with women more likely to receive visitors in NHs than men.

## Discussion

This study described the socio-economic, health and nutritional status of elderly people residing in Lebanese NHs and compared these characteristics across gender. The findings generally showed that all elderly in general and women in particular suffered from low socio-economic status and poor health conditions.

### Nutritional status

Based on the MNA scores, 3.2% of elderly were malnourished while 27.6% were at risk of malnutrition (see for instance [21]). These results were within or lower than the ranges reported in international studies [12]. The results from 32 multinational studies including 6,821 institutionalized elders indicated that the prevalence of malnutrition and its risk were 5-71% and 27-70% respectively [12], while a recent study conducted among 895 Spanish elders residing in 34 NHs found the prevalence of malnutrition and its risk were 2.8% and 37.3% respectively [35].

In comparison to community-dwelling elderly, several studies reported a high prevalence of malnutrition among elderly residing in NHs [3,12,13]. In Lebanon, the prevalence of both malnutrition and at risk of malnutrition among rural free elders living in their natural homes was 8% and 29.1% respectively [7]. Moreover, in 21 studies carried in several countries among 14,149 participating free elders, the prevalence of both malnutrition and at risk of malnutrition were 2% and 24% respectively [12].

The reason for the relatively high nutritional levels among elderly participating in this study in comparison to international levels was the exclusion of those elders who were severely ill, blind, deaf and/or demented. This latter sub-group was more susceptible to malnutrition and adverse health conditions.

In our study, the most important finding was the no-significant difference between men and women on nutritional level. Similar finding was reported by Tsai *et al.* when using MNA to assess the prevalence of malnutrition among institutionalized and non-institutionalized Taiwanese elderly with 2.4% for women and 1.7% for men [13]. However, other studies carried out in different settings reported gender differences [3,4,7]. In Lebanon, the prevalence of malnutrition among rural community-dwelling elderly was 9.1% for women and 6.9% for men [7]. In Italy, this prevalence among all elderly, institutionalized and non-institutionalized was 26% and 16.3% among women and men respectively [29]. Moreover, Vandewoude and Van Gossum, after assessing and comparing nutritional status between community dwelling and nursing home elderly, found that the prevalence of malnutrition was higher among women, nursing home elderly and old age groups [4].

According to the BMI score, 20% of participants were classified as obese and obesity frequency was two times higher among elderly women than men. In Lebanon, similar results were reported in a small sample of institutionalized elders [10] and the frequency of obesity was greater among free elderly women than men living in both rural and urban regions [7,36]. Furthermore, 16% of elderly participating in this study were classified as malnourished and might suffer from adverse health outcomes [37-39].

### Socio-economic status

Regarding the socio-economic status, women were significantly less educated than men and more likely to have low occupational status, to be single or widowed and to rely on their children and relatives. These findings were corroborated by a number of studies in Lebanon and the Arab countries [6-9,11]. In the Arab world illiteracy among women was higher than men [11]. In Lebanon, it has become less of a problem. School enrollment rates showed an increase at both the elementary and secondary educational levels reaching in 2009 77.4% and 85.2% for men and women respectively [40]. Given this trend, the gender based educational gap could significantly diminish in the near future and thus trigger a dynamic process of drawing more women to the workforce and achieving more economic equality with men. Second, the low occupational level of women in comparison to men could be explained by their low educational level and weak participation in the labor force. Culturally, women In the Middle East region play the traditional role of homemaker; caring for her children while men whether a husband, son, or relative, play the provider role of securing financial and material support [11]. The homemaker position could explain the low physical activity among women and their financial dependence on children and relatives. Third, women debilitating economic factors were reflected in later life experience of widowhood.

Furthermore, the Lebanese lawmakers set the retirement age at 64. After retirement, most elders would lose part of their income and health benefits and become dependent financially and socially on their children and relatives. Research data have shown that limited social support was associated with poor health outcomes [41]. With the family close-knit social structure becoming tattered because of the immigration of young adults and the more participation of female in the labor force, elders are left with little or no social support and subsequently adverse health effects. The Lebanese Government policies neglected the new socio-demographic realities. New policies are needed to address this issue and that stress on bringing elderly back to partially work. With Elders reintegrated in the workforce or provided minimal work (related activities) they would experience less depression and other related diseases [42,43] and thus would improve their living conditions in NHs [19,21,44].

In addition, Lebanese elderly men were likely to exhibit smoking behaviors more than women [11] and to develop smoking related diseases leading to early mortality.

### Health conditions

In our study, most elderly suffered from chronic non-communicable diseases, functional impairment and depressive symptoms with no significant difference between men and women but with higher prevalence of chronic diseases and functional impairment among women than men. Similar findings were reported in other studies conducted in Lebanon [6,7]. Women tended to get sicker than men as they lived longer with non-fatal illnesses [5]. Regarding depression, 60% of the elderly participating in this study, had some degree of depression with no differences between men and women. Similar results were reported by a pilot study that included 102 elders living in three different Lebanese NHs [20]. The high prevalence of depressive symptoms among institutionalized elders could be reduced by improving their nutritional status [45], their belief in a just world [23] and their quality of life [21].

### Limitations

This research had two major limitations. The first limitation was related to the small number of elderly who agreed to participate, had the cognitive functioning, and functional ability to interact with researchers. The second limitation was related to instrument validity. The MNA instrument used had not been validated among the Lebanese population and thus caution must be approached when generalizing about the nutritional outcomes resulting from this study. We recommend for future work a replication of this study using a larger sample of elderly from Lebanon and the Middle East.

### Strengths

This study is quite original on three different accounts. It is the first study performed nationwide covering the largest number of NH institutions in various locations, stewardships, and with diverse elderly residents’ backgrounds. Second, it is the first study that assessed all these factors: demographic, socio-economic, functional, psychological and nutritional status, among elderly residing in Lebanese NHs. Lastly, it is the first study in Lebanon that compared all these characteristics across gender.

### Recommendations

Currently, both Ministries of Social Affairs and Public Health are focusing on acute care and fatal illnesses [46]. Their programs and policies do not cover elderly healthcare provisions or those suffering from chronic illnesses. Working on the belief that better health is associated with better socioeconomic status, this study appeals for new policies that support elderly with overall subsidized government healthcare. It also appeals to policies pertaining to elderly paid work plans and the institutionalization of new healthcare policies and standards. Currently, there are no valid operational standards to evaluate NHs in Lebanon. The setting of standards and policies for NHs may contribute to the wellbeing and wellness of institutionalized elderly. For instance, screening elderly for cognitive function, depression, malnutrition and autonomy on admission to the NHs and early intervention may prevent health deterioration and facilitate successful aging. Finally, it is high time to address the issue of gender discrepancy as societies with large gender disparity will likely affect the health of both men and women [47].

## Conclusion

The results in the present study were unique in highlighting differences between gender on nutritional, functional and psychological status among elderly residing in Lebanese NHs. The findings showed that most elderly had poor socioeconomic and health status with women being the most disadvantaged. This study raised issues of elderly care in Lebanon. It encouraged health providers and government agencies to improve elderly quality of life. It called for a nationwide awareness campaign to promote health among all elderly residing in NHs with more attention giving to women wellbeing.

## Competing interests

The authors declare that they have no competing interests.

## Authors’ contributions

JD conceptualized the study presented in this paper; administered the questionnaires; collected, analyzed, interpreted the data and wrote the paper. RN has planned, operationally ran and wrote part of the study. DH co-wrote the paper. All authors have read and approved the final manuscript.

## Pre-publication history

The pre-publication history for this paper can be accessed here:

http://www.biomedcentral.com/1471-2458/14/629/prepub
